# Evaluation of an electronic consultation service for transgender care

**DOI:** 10.1186/s12875-021-01401-3

**Published:** 2021-03-20

**Authors:** Jatinderpreet Singh, Allison Lou, Michael Green, Erin Keely, Mary Greenaway, Clare Liddy

**Affiliations:** 1 Department of Family Medicine, Queen’s University, Kingston, ON Canada; 2 C.T. Lamont Primary Health Care Research Centre, Bruyère Research Institute, Ottawa, ON Canada; 3 Sherbourne Health, Toronto, ON Canada; 4 Department of Public Health Sciences, Queen’s University, Kingston, ON Canada; 5 Department of Medicine, University of Ottawa, Ottawa, ON Canada; 6 Division of Endocrinology/Metabolism, The Ottawa Hospital, Ottawa, ON Canada; 7 HealthSource Medical Clinic, Toronto, ON Canada; 8 Department of Family Medicine, University of Ottawa, Ottawa, ON Canada

**Keywords:** Transgender care, Electronic consultation, eConsult, Primary care, Access to care, Specialist care

## Abstract

**Background:**

Access to transgender care in Canada is poor. Although primary care providers are ideally positioned to initiate care, many feel uncomfortable providing transgender care. This study aimed to explore the impact of an electronic consultation (eConsult) service between primary care providers and transgender care specialists on access to care and to explore the content of clinical questions that were asked.

**Methods:**

This was a retrospective mixed methods analysis of 62 eConsults submitted between January 2017 and December 2018 by primary care providers to specialists in transgender care in a health region in eastern Ontario, Canada. A descriptive analysis was conducted to assess the average response time and the total time spent by the specialist for the eConsults. An inductive and deductive content analysis was carried out to identify common themes of clinical questions being asked to transgender specialists. A post-eConsult survey completed by primary care providers was assessed to gain insight into avoided face-to-face referrals and overall provider satisfaction.

**Results:**

The median specialist response time was 1.2 days (range: 1 h to 5 days) and the average time spent by specialists per eConsult was 18 min (range: 10 to 40 min). The qualitative analysis identified six major themes: 1) interpretation/management of abnormal bloodwork, 2) change in management due to lack of desired effect/hormone levels not a target, 3) initiation of hormone therapy/initial work up, 4) management of adverse effects of hormone therapy, 5) transition related surgery counseling and post-op complications, and 6) management of patients with comorbidities. Approximately one-third of eConsults resulted in an avoided face-to-face referral and 95% of primary care providers rated the value of their eConsult as a 5 (excellent value) or 4.

**Conclusions:**

This study demonstrated that a transgender eConsult service has potential to significantly improve access to care for transgender patients. Given the importance that timely access has on improving mental health and reducing suicide attempts, eConsult has the potential to make a substantial clinical impact on this population. Identified themes of eConsult questions provides insight into potential gaps in knowledge amongst primary care providers that could help inform future continuing education events.

## Background

Consistent evidence has demonstrated that transgender individuals are at increased risk of poor mental and physical health outcomes [1–5]. Studies conducted in Canada have shown that trans people are at an increased risk of gender-based discrimination, verbal harassment, and physical and sexual abuse [2, 6]. Transgender individuals have been shown to have higher rates of anxiety and depression relative to cis-gender individuals [3–6]. More concerning is that there is a significantly higher likelihood of a suicide attempt in transgender individuals who want to transition but have not initiated the process [1, 2, 6]. A recent Ontario study found that 43% of transgender individuals had attempted suicide, with 10% having an attempt within the past year [2]. Evidence shows that the rate of suicide attempts significantly decreases once hormone or surgical transition is initiated, which demonstrates the importance of timely access to transgender care [2, 7].

Unfortunately, access to transgender care in Canada has historically been poor [8]. Although primary care providers are ideally positioned to initiate hormone therapy and provide transgender care, evidence shows that many feel uncomfortable providing transgender care, due in part to a lack of training [9, 10]. As a result, many patients seeking transition care are referred to specialists, which further delays the onset of care.

Electronic consultation is a promising approach to improve timely access to specialist care. The Champlain BASE™(Building Access to Specialists through eConsultation) electronic consult (eConsult) service was developed in Ottawa, Ontario in 2010 to address excessive specialist wait times. The Champlain BASE™ eConsult service is a secure online platform that facilitates asynchronous communication between primary care providers and specialists [11]. Through eConsults, primary care providers are able to submit questions (along with pertinent lab results, images, etc.) to specialists, who in turn are able to provide timely recommendations or confirm the need for a face-to-face referral [11]. Studies conducted to date assessing the Champlain BASE™ eConsult service have demonstrated significant improvements in access to specialty care, a reduced need for face-to-face specialty visits, and high levels of provider and patient satisfaction [11–17]. Recent studies have also demonstrated that eConsult is both safe and results in overall cost savings [11–18]. Furthermore, several studies have pointed to the unique learning opportunities that users have noted through using the system and several researchers have highlighted the potentially rich learning opportunities that could be gained from continuing education sessions focussed on the questions and content of eConsults [19, 20].

The aim of this study is to examine the impact of an electronic consultation service on improving access to transgender care as measured by the wait time and the number of traditional face-to-face referrals avoided. Also, this study aims to explore and characterize the content of clinical questions being asked to transgender specialists through eConsult.

## Methods

This is a mixed methods analysis of 62 eConsults submitted between January 2017 and December 2018 by primary care providers to specialists in transgender care. This study received ethics approval from the Queen’s Health Sciences and Affiliated Teaching Hospital Research Ethics Board.

### Participants and setting

Participants in the study included primary care providers who were registered to the Champlain BASE™ eConsult service and who initiated at least one electronic consultation between January 2017 and December 2018 to a transgender specialist.

The Champlain BASE™ eConsult service is based in the Champlain region of Ontario, Canada. This region is home to approximately 1.3 million residents and encompasses Ottawa and surrounding cities and rural communities. The region is both linguistically and culturally diverse, with one in five residents speaking French and one in six residents speaking a language other than French or English [21].

Transgender care in Ontario is largely embedded in general primary care practice. Family physicians and nurse practitioners (in group or solo practices) who feel comfortable providing transgender care and feel it is within their scope manage hormone therapy and surgery referrals. Primary care is supported by endocrinologists and psychiatry through specialty clinics (eg, The Centre for Addiction and Mental Health Gender Identify Clinic) in terms of diagnoses and recommending treatments.

### Champlain BASE™ eConsult service

The Champlain BASE™ eConsult system is an asynchronous secure online platform that facilitates communication between a primary care provider and a specialist [11, 12]. The system is funded through the Ministry of Health and Long-Term Care of Ontario.

**Fig. 1:**
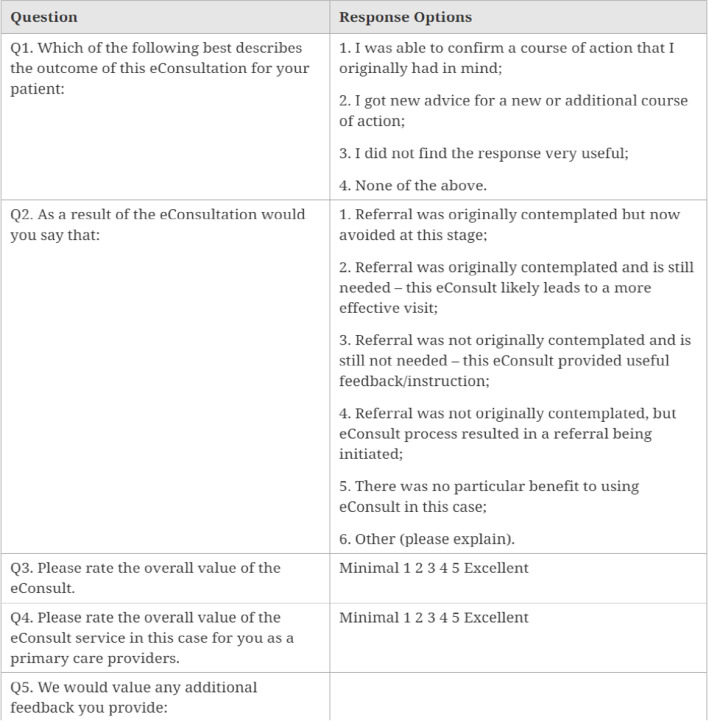
Close-out primary care provider survey

A primary care provider initiates an eConsult by logging onto the secure web portal and filling out a structured electronic form that includes the patient’s medical history and the clinical question being asked [11, 12]. Any pertinent attachments such as labs or images can also be included with each eConsult, if required. Once an eConsult is submitted, it is assigned to one of the specialists in transgender care based on availability. The specialist can request additional information, provide advice, or advise for a face-to-face consultation [11, 12]. Once a case is closed, the primary care provider is prompted to complete a close-out survey (Fig. 1) that gathers information on the actions taken following the consultation and the physicians overall satisfaction with the process [11, 12].

### Study design

This is a retrospective mixed methods analysis of the 62 eConsults. A descriptive analysis of time stamps for each eConsult was completed to assess the response time and total time spent by the specialist to complete the eConsult.

An inductive and deductive content analysis of the primary care provider eConsult questions was also conducted to gain insight into common themes [12, 19, 22]. Two investigators, AL (transgender care specialist), and JPS (family medicine resident), independently reviewed clinical questions being asked by primary care providers using a generalized validated taxonomy and also open coding to categorize questions not captured by the framework [23]. Both investigators discussed their independent assessments using an iterative approach to come to a consensus on a final list of themes. In many cases, a single eConsult had multiple questions, and thus, each eConsult was not restricted to only one theme to ensure no information was lost. In total, 20 eConsults were assessed until saturation was achieved. Once themes were identified, a single investigator (JPS) completed a deductive analysis and went through and coded the remaining 42 eConsults. No new themes emerged from the final 42 eConsults. Themes were externally validated by the remainder of the team (2 family physicians, 1 transgender specialist, and an endocrinologist).

All eConsult close-out surveys (Fig. 1) done by primary care providers were assessed to gain insight into the course of action taken following the consult, provider satisfaction, and to quantify the proportion of face-to-face specialist visits that were avoided.

## Results

Between January 2017 and December 2018 there were a total of 62 eConsults that were submitted to a transgender specialist in the Champlain BASE™ electronic system. These were submitted by 37 unique primary care providers, of which, 26 (70%) were physicians and 11 (30%) were nurse practitioners. In terms of patient characteristics, the average age of the patients was 30 (range: 16 to 62) years of age and 65% of patients identified as a trans-woman (*n* = 40).

The median time for a specialist to respond to the eConsult was 1.2 days, with the quickest response being in one hour and the longest being after five days. The average time the specialist spent in responding to each eConsult was 18 min (range: 10 to 40 min).

### Identified themes from content analysis

**Fig. 2:**
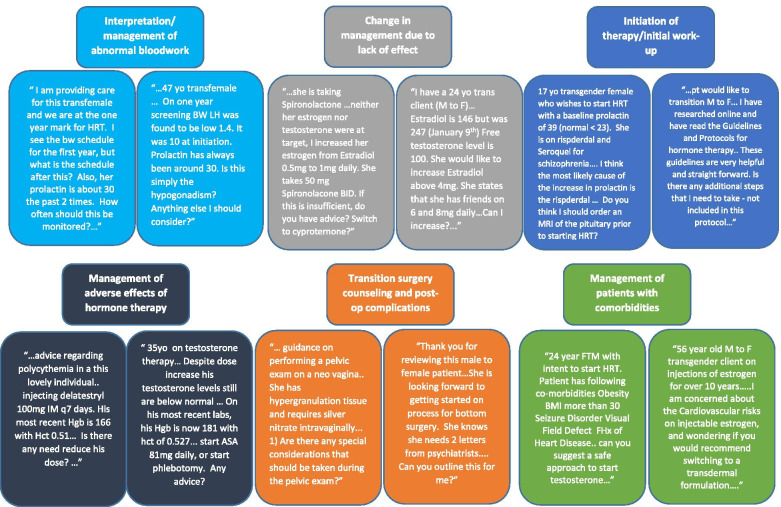
Six identified themes from qualitative analysis

The qualitative analysis of the questions raised by the primary care providers resulted in six main themes: 1) interpretation/management of abnormal bloodwork, 2) change in management due to lack of desired effect/hormone levels not a target, 3) initiation of hormone therapy/initial work up, 4) management of adverse effects of hormone therapy, 5) transition related surgery counseling and post op complications, and 6) management of patients with comorbidities. Figure 2 summarizes each of the themes with quotes from sample eConsults. The most common topic that was identified was related to questions involving the interpretation and management of abnormal bloodwork findings (29%, *n* = 18), which was present in about one-third of eConsults (Table 1). The questions varied from getting guidance on how to manage testosterone and estradiol levels that were not within the desired range to other hormones such as prolactin and luteinizing hormone being out of range. Another question that came up in several eConsults (*n* = 3) was related to the management of polycythemia in patients on testosterone therapy.

**Table 1:** List of content topics identified in eConsults

eConsult content topic	# of eConsults	% of eConsults
Hormone therapy monitoring bloodwork (interpretation/management of abnormal findings)	18	29
Hormone therapy dose adjustments	9	15
Medication delivery method (oral, patch, etc.)	9	15
Initiating hormone therapy (therapy, initial work-up)	8	13
Management of adverse effects of hormone therapy	8	13
Management of patients with comorbidities	6	10
Management of transition surgery post-op complications	5	8
Hormone therapy: Medication change due to lack of effect	5	8
Transition surgery (Counseling/approval process)	5	8
Community resources (Clinical/non-clinical)	3	5
Preventive care (eg, pap, mammogram, etc.)	2	3
Non-clinical (eg. Switching gender on licence)	1	2

The next most common topics were related to hormone therapy dose adjustments (*n* = 9, 15%) and questions related to alternative delivery methods (*n* = 9, 15%) of hormone therapy (eg, oral, patch, etc.). The former most often related to the need for dose adjustments due to a lack of clinical effect or levels of testosterone or estradiol being out of range. Other common topics included management of adverse effects of hormone therapy (*n* = 8, 13%), management of hormone or surgical related issues in transgender patients with medical and/or mental health comorbidities (*n* = 6, 10%), and management of post-op complications of transition-related surgery (*n* = 5, 8%).

### Post-eConsult survey

#### Course of action following eConsult

**Fig. 3:**
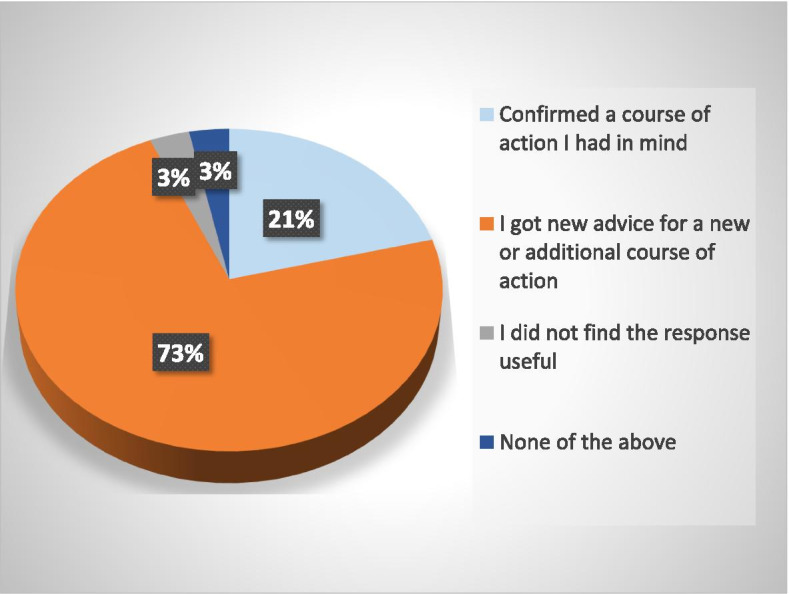
Primary care provider responses to the close out survey assessing the outcome of the eConsult

**Fig. 4:**
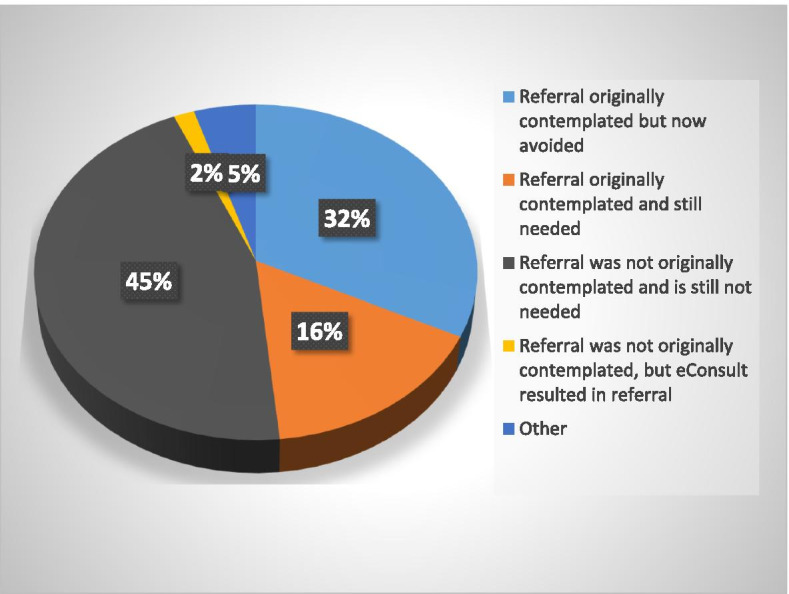
Primary care provider responses to the close out survey assessing the course of action following the eConsult and the need for a face to face referral

In approximately three-quarters of the eConsults (Fig. 3), primary care providers responded that they got new advice for a new or additional course of action, while 21% felt the eConsult confirmed a course of action they had previously contemplated. Furthermore, in about one-third of cases (32%, *n* = 20), the primary care provider responded that they had initially contemplated a face-to-face referral, however, found it was not necessary following the eConsult (Fig. 4). In 45% of cases, a referral was not initially contemplated and was still not necessary following the eConsult, whereas in 16% (*n* = 10) of cases, a referral was initially contemplated and still needed following the eConsult.

Primary care providers were asked to rate the overall value of the eConsult to their patient on a Likert scale from 1 (minimal value) to 5 (excellent value). In total, 79% (*n* = 49) of cases were given a 5 and 16% (*n* = 10) were rated as a 4. No providers reported minimal value for their eConsult. The comments left by primary care providers reflected their appreciation for the prompt response and useful recommendations. For example,

*“I truly appreciate your recommendations and references. This was truly helpful.”*

*“Thank you for your prompt reply and useful recommendations. Very helpful.”*

In addition, there were several comments among primary care providers that reflected the need for further training and education in the area of transgender care. For example,

“trans healthcare CME (continuing medical education) is +++++++ needed!”

When asked to similarly rate the overall value of the eConsult service as a whole to their primary care practice, the majority (75%) responded with either a 4 (33%, *n* = 21) or 5 (42%, *n* = 26).

## Discussion

This study demonstrated that a transgender eConsult service has the potential to significantly improve access to care for transgender patients by reducing wait times for specialist advice from months to days. Given the importance that timely access has on improving mental health and reducing suicide attempts, eConsult has the potential to make a substantial clinical impact on this population [2]. The qualitative assessment of eConsults demonstrated that the most frequently asked questions related to interpretation and management of abnormal blood work and recommendations on changing the management plan to address a lack of a clinically desired effect. Other common themes identified included initiation of hormone therapy/initial work up, management of adverse effects of hormone therapy, transition related surgery counseling and post op complications, and management of patients with comorbidities.

Few studies have examined the impact of an electronic consultation system on improving access to transgender care [24]. A US based study conducted by Shipherd et al. examined an interdisciplinary electronic consultation service focused on transgender care for Veterans. This was a nationwide service where consults were submitted to a team of providers (eg, primary care physician, psychiatrist, psychologist, social work, pharmacist) who could each provide their feedback on a given consult [24]. The most common themes related to the questions being asked through electronic consultation related to hormone therapy and medications, primary care comorbidity and screening, mental health evaluations and psychotherapy. These findings are in line with this study aside for questions focussed on mental health and psychotherapy. This difference may potentially be due in part to the difference in populations as the study conducted by Shipherd et al. only involved transgender veterans whose mental health needs likely differ from those of the general transgender population [24]. Also, consults in the current study were being addressed by a primary care physician with expertise in transgender care, whereas the Veterans study involved a multi-disciplinary team including a psychiatrist. As such, it is possible that potential consults related to gender dysphoria or other mental health concerns are potentially being directed to a psychiatrist through the eConsult system in the Champlain region versus the Veterans study, which had a psychiatrist on the team assessing consults [24].

The median response time for eConsults was 1.2 days, which provides insight into the tremendous potential of this platform to improve access to transgender care. Also, about one third of eConsults resulted in an avoided face-to-face referral. As such, eConsult also has the potential to free up space for those who actually require a face-to-face referral and may decrease the overall wait-time for patients. The average specialist response time and percentage of avoided unnecessary face-to-face referrals is in-line with other eConsult studies that have looked at other specialties [11–13, 17]. The wait time for transgender specialty care in Canada is long, with gender identity specialist clinics reporting wait times of upwards of 8 to 9 months for an initial assessment [25]. The improved access through eConsult is promising as previous studies have shown the important impact that more timely access to transition care can have on clinical outcomes for transgender patients [1]. For example, previous studies have reported that the lifetime prevalence of a suicide attempt in someone that is transgender is 46% versus 3.8% in someone that is cis-gender [2, 6, 7, 26]. A UK study of trans people found that a completed medical transition greatly reduced rates of suicidal ideation and attempts relative to those at different stages of transition [1]. The study also reported that 67% of transitioning people thought about suicide prior to transitioning versus only 3% after their transition.

Previous studies have highlighted the potential educational value of eConsults for primary care providers. For example, a qualitative analysis conducted by Liddy et al., found that many providers using the system have commented on the educational value of specialist responses, which often times extends beyond the specific case-related question [20]. Amongst the eConsults assessed, there were a number of recurring themes with questions across different providers, potentially representing common gaps in knowledge amongst primary care providers. We feel that the specific identified themes and cases in this study could form the basis of a continuing educational session that would be of great value to primary care providers. This information would also be beneficial for organizations who have developed guidelines for transgender care to support them in making refinements to address ongoing gaps in knowledge. This would be of particular benefit in the area of transgender care as previous studies have identified a lack of comfort amongst primary care providers with transgender care and a gap in current training for new residents [9, 10]. For example, a Canadian-based study demonstrated that only 10% of family medicine residents reported that they had the competency to confidently provide transgender care in their practices [9]. Future work will focus on designing and evaluating a continuing education session on transgender care, which attempts to incorporate sample cases through eConsult to address recurring gaps in knowledge.

### Study limitations

This study has several limitations. Firstly, the data collected for this study was from a single health region in Ontario. Although the Champlain region is diverse, this limits the generalizability of our findings. That being said, the results from this study were externally validated by members of our team who are transgender specialists and perform eConsults for patients across the province of Ontario. Based on their experience, they found the identified themes to be comparable to other regions in the province of Ontario. Also, as mentioned above, few studies have looked at transgender eConsultations in other regions, which limits our ability to compare our findings to other jurisdictions. Furthermore, we have limited follow up data and are unable to ascertain if advice from a particular eConsult led to an adverse event or a future in-person referral. In addition, since this was a retrospective analysis of pre-existing data, we were unable to dictate what information was being collected from primary care providers in the exit survey. For example, in 45% of eConsults the primary care providers stated that they did not contemplate a face to face referral prior to submitting their eConsult and did not require one following the eConsult. It would have been interesting to gain insight into what their plan would have been had eConsult not existed. This would be of particular interest as three quarters of all providers stated they obtained useful information that altered their management plan following the eConsults. Lastly, this study did not assess the perspectives and acceptability of eConsult amongst the transgender specialists. That being said, Keely et al. have previously reported specialist perspectives of active participants on the eConsult platform used in this study and found that over 90% felt the service was a feasible way to improve access to specialist care and could be integrated into their clinical workflow without difficulty [27].

## Conclusions

This study demonstrated that a transgender eConsult service has the potential to significantly improve access to care for transgender patients. Also, with one third of eConsults resulting in an avoided face to face referral, this system may have the potential to free up space for more urgent cases that require a face-to-face referral to be seen in a timely manner. Given the importance that timely access has on improving mental health and reducing suicide attempts, eConsult has the potential to make a substantial clinical impact on this population [2]. Furthermore, this study highlighted six key themes of eConsult questions that provide insight into potential gaps in knowledge amongst primary care providers. This information could help in guiding future continuing education events or provide guidance in refining current guidelines to help address current knowledge gaps.

Future studies will aim to study the value of a continuing education event guided by common gaps in transgender care identified through eConsult cases. Furthermore, it would be of interest to conduct patient and specialist interviews to understand their experiences with eConsult and to gain a better appreciation of the benefits of such a tool from their perspectives. Lastly, studying the impact of improved access to care through eConsult on clinical outcomes such as suicidal ideation and attempts will be examined in future work.

## Data Availability

The datasets used and/or analyzed during the current study are available from the corresponding author on reasonable request.

## Abbreviations

BASE™: Building Access to Specialists through eConsultation
CME: Continuing Medical Education
eConsult: Electronic consultation

## References

[1] Bailey L, Ellis S, Mcneil J (2014). Suicide risk in the UK Trans population and the role of gender transition in decreasing suicidal ideation and suicide attempt. Ment Health Rev J.

[2] Bauer GR, Pyne J, Francino M, Hammond R (2013). Suicidality among trans people in Ontario: implications for social work and social justice. Rev Serv Soc.

[3] Reisner SL, Poteat T, Keatley J, Cabral M, Mothopeng T, Dunham E (2016). Global health burden and needs of transgender populations: a review. Lancet Lond Engl.

[4] Rotondi NK, Bauer GR, Travers R, Travers A, Scanlon K, Kaay M (2011). Depression in male-to-female transgender Ontarians: results from the trans PULSE project. Can J Commun Ment Health.

[5] Veale JF, Watson RJ, Peter T, Saewyc EM (2017). Mental health disparities among Canadian transgender youth. J Adolesc Health.

[6] Bauer GR, Scheim A. Transgender people in Ontario, Canada: statistics to inform human rights policy [Internet]. London; 2015. Available from: https://transpulseproject.ca/wp-content/uploads/2015/06/Trans-PULSE-Statistics-Relevant-for-Human-Rights-Policy-June-2015.pdf.

[7] Bell J, Purkey E (2019). Trans individuals’ experiences in primary care. Can Fam Phys.

[8] Frohard-Dourlent H, Coronel V, Saewyc E. A survey of experiences with surgery readiness assessment and gender affirming surgery among trans people in Ontario. Vancouver; 2018. (Stigma and Resilience Among Vulnerable Youth Centre).

[9] Coutin A, Wright S, Li C, Fung R (2018). Missed opportunities: are residents prepared to care for transgender patients? A study of family medicine, psychiatry, endocrinology, and urology residents. Can Med Educ J.

[10] McPhail D, Rountree-James M, Whetter I (2016). Addressing gaps in physician knowledge regarding transgender health and healthcare through medical education. Can Med Educ J.

[11] Archibald D, Stratton J, Liddy C, Grant RE, Green D, Keely EJ. Evaluation of an electronic consultation service in psychiatry for primary care providers. BMC Psychiatry. 2018;18(1):119 e1–e7. 10.1186/s12888-018-1701-3 PMC5932827 29720133

[12] Chan E, Johnson CB, Liddy C, Keely E, Gauthier N, Turek M, et al. Paging the eCardiologist: insights into referral behaviour of primary care physicians from qualitative analysis of a cardiology eConsult service. Digit Health. 2018;4:e1–e9. 10.1177/2055207618792140 PMC6120168 30186618

[13] Keely E, Canning S, Saloojee N, Afkham A, Liddy C. Improving access to gastroenterologist using eConsultation: a way to potentially shorten wait times. J Can Assoc Gastroenterol. 2018;1(3):124–8. 10.1093/jcag/gwy017 PMC6507275 31294353

[14] Liddy C, McKellips F, Armstrong CD, Afkham A, Fraser-Roberts L, Keely E (2017). Improving access to specialists in remote communities: a cross-sectional study and cost analysis of the use of eConsult in Nunavut. Int J Circumpolar Health.

[15] Liddy C, Moroz I, Afkham A, Keely E (2017). Evaluating the implementation of the Champlain BASE^TM^eConsult service in a new region of Ontario, Canada: a cross-sectional study. Healthc Policy Polit Sante.

[16] Liddy C, Moroz I, Mihan A, Nawar N, Keely E (2019). A systematic review of asynchronous, provider-to-provider, electronic consultation services to improve access to specialty care available worldwide. Telemed J E-Health.

[17] Witherspoon L, Liddy C, Afkham A, Keely E, Mahoney J (2017). Improving access to urologists through an electronic consultation service. Can Urol Assoc J.

[18] Liddy C, Moroz I, Keely E, Taljaard M, Mark Fraser A, Deri Armstrong C (2018). The use of electronic consultations is associated with lower specialist referral rates: a cross-sectional study using population-based health administrative data. Fam Pract.

[19] Archibald D, Liddy C, Lochnan HA, Hendry PJ, Keely EJ (2018). Using clinical questions asked by primary care providers through eConsults to inform continuing professional development. J Contin Educ Health Prof.

[20] Liddy C, Abu-Hijleh T, Joschko J, Archibald D, Keely E (2019). eConsults and learning between primary care providers and specialists. Fam Med.

[21] Ministry of Health and Long term Care. Champlain LHIN [Internet]. Available from: http://www.champlainlhin.on.ca/AboutUs/Intro.aspx.

[22] Silverman D (2000). Doing qualitative research.&nbsp;A practical handbook.

[23] Ely JW, Osheroff JA, Gorman PN, Ebell MH, Chambliss ML, Pifer EA (2000). A taxonomy of generic clinical questions: classification study. BMJ.

[24] Shipherd JC, Kauth MR, Matza A (2016). Nationwide Interdisciplinary E-consultation on transgender care in the Veterans health administration. Telemed J E-Health.

[25] CAMH. Gender identity clinic [Internet]. [cited 13 Jan 2021]. Available from: www.camh.ca/en/your-care/programs-and-services/gender-identity-clinic-adult.

[26] Weissman MM, Bland RC, Canino GJ, Greenwald S, Hwu HG, Joyce PR (1999). Prevalence of suicide ideation and suicide attempts in nine countries. Psychol Med.

[27] Keely E, Williams R, Epstein G, Afkham A, Liddy C (2019). Specialist perspectives on Ontario provincial electronic consultation services. Telemed J E-Health.

